# A novel technique to quantify the instantaneous mitral regurgitant rate

**DOI:** 10.1186/1532-429X-15-74

**Published:** 2013-08-31

**Authors:** Seth Uretsky, Farooq A Chaudhry, Linda Gillam, Srinivasa Gurram, Sri Lakshmi Kala Bonda, Harikrishna Ponnam, Eric Bader, Naganath Thota, Randy Cohen, Azhar Supariwala, Steven D Wolff

**Affiliations:** 1 Department of Medicine, Division of Cardiology, St. Luke’s-Roosevelt Hospital Center, 1111 Amsterdam Ave, Cardiology 3rd Floor, New York, NY 10025, USA; 2 Department of Cardiology, Mount Sinai School of Medicine, New York, NY USA; 3 Department of Cardiovascular Medicine, Morristown Medical Center, Morristown, NJ USA; 4 Carnegie Hill Radiology, New York, NY USA

**Keywords:** Mitral regurgitation, Cardiovascular magnetic resonance, Echocardiography, Temporal variation

## Abstract

**Background:**

The systolic variation of mitral regurgitation (MR) is a pitfall in its quantification. Current recommendations advocate using quantitative echocardiographic techniques that account for this systolic variation. While prior studies have qualitatively described patterns of systolic variation no study has quantified this variation.

**Methods:**

This study includes 41 patients who underwent cardiovascular magnetic resonance (CMR) evaluation for the assessment of MR. Systole was divided into 3 equal parts: early, mid, and late. The MR jets were categorized as holosystolc, early, or late based on the portions of systole the jet was visible. The aortic flow and left ventricular stroke volume (LVSV) acquired by CMR were plotted against time. The instantaneous regurgitant rate was calculated for each third of systole as the difference between the LVSV and the aortic flow.

**Results:**

The regurgitant rate varied widely with a 1.9-fold, 3.4-fold, and 1.6-fold difference between the lowest and highest rate in patients with early, late, and holosystolic jets respectively. There was overlap of peak regurgitant rates among patients with mild, moderate and severe MR. The greatest variation of regurgitant rate was seen among patients with mild MR.

**Conclusion:**

CMR can quantify the systolic temporal variation of MR. There is significant variation of the mitral regurgitant rate even among patients with holosystolic MR jets. These findings highlight the need to use quantitative measures of MR severity that take into consideration the temporal variation of MR.

## Background

Echocardiography is the most commonly used imaging modality to assess mitral regurgitation [1,2]. Several echocardiographic parameters allow for the quantification of mitral regurgitation [1-3]. An important consideration when quantifying mitral regurgitation is the dynamic nature of the regurgitation and the temporal variation of the regurgitant volume throughout systole [4-7]. The American Society of Echocardiography recommendations for evaluation of the severity of native valvular regurgitation highlight the importance of accounting for the systolic temporal variation of mitral regurgitant severity and the most commonly used echocardiographic parameters account for this temporal variation [2]. While prior studies have qualitatively described the various patterns of the systolic temporal variation of mitral regurgitation, no study has quantified it [2,4,5].

Cardiovascular Magnetic Resonance (CMR) can be used to quantify mitral regurgitation severity [8-10]. Using CMR we have developed a novel method to quantify the instantaneous mitral regurgitant rate. The purpose of this study is to:1) assess the feasibility of this method in patients with mitral regurgitation, 2) to quantify the instantaneous mitral regurgitant rate, and 3) describe the patterns of temporal variation of mitral regurgitation as a function of systole.

## Methods

### Patients

This prospective study included 41consecutive patients (mean age 57 ± 15 yrs, male 56%) who underwent CMR evaluation for the assessment of mitral regurgitation. Exclusion criteria included greater than mild aortic regurgitation or stenosis, greater than mild mitral stenosis, the presence of an intracardiac shunt, hypertrophic cardiomyopathy, pregnancy, or a contraindication to CMR. Patient baseline clinical characteristics and symptoms were collected at the time of enrollment in the study. This research protocol was approved by the Institutional Review Board of St. Luke’s-Roosevelt Hospitals and all patients gave their informed consent to participate in this study.

### Cardiovascular magnetic resonance

CMR studies were performed with a 1.5 Tesla MR scanner using an 8-element, phased-array coil (GE Signa, EXCITE, GE Medical Systems, Milwaukee, Wisconsin, USA). Images were acquired with ECG gating and breath holding. Short and long axis cine images were acquired using a steady-state free precession pulse sequence (FIESTA) with the following parameters: TR/TE 3.3 ms/1.4 ms, 20 views per segment, FOV 35 × 35 cm, acquisition matrix 192 × 160, slice thickness 8 mm, slice gap 0 mm, flip angle 45 degrees, receive bandwidth 125 kHz. Phase contrast images were acquired perpendicular to the proximal pulmonary artery and perpendicular to the proximal aorta to quantify flow in these vessels using the following parameters: TR/TE 7.5 ms/2.9 ms, 6 views per segment, Venc 250 cm/s, FOV 35 × 35 cm, acquisition matrix 256 × 128, slice thickness 4 mm, flip angle 20 degrees, receive bandwidth 31.3 kHz. After the clinical scan was completed, additional phase contrast images were acquired of a stationary bottle of water (phantom) for baseline flow correction [11]. Images were reviewed and analyzed using SuiteHeart software (NeoSoft, Pewaukee, Wisconsin). Left ventricular volumes were determined using the automated left ventricular segmentation algorithm which excludes papillary muscles and trabeculations from the left ventricular cavity and which uses a long axis image to define the position of the base of the left ventricle. Right ventricular volumes were determined by manual segmentation of the short axis images. Aortic and pulmonary artery flow values were determined using the resident semi-automated algorithm. Correction for baseline offsets was performed using a phantom phase contrast image as described previously [11]. For each patient, two or three flow acquisitions were made and the flow values were averaged. Mitral regurgitant volume was determined as the difference between the left ventricular stroke volume (as determined by endocardial segmentation) and forward flow as previously described [10]. MR was categorized as per the AHA/ACC guidelines: mild < 30 ml, moderate 30-59 ml, and severe ≥60 ml. The reproducibility of this method has been previously published [10]. Leaflet prolapse was defined as leaflet excursion of ≥ 2 mm beyond the mitral valve annulus in the 3-chamber view [1].

### Determination of mitral regurgitant jet type

**Figure 1:**
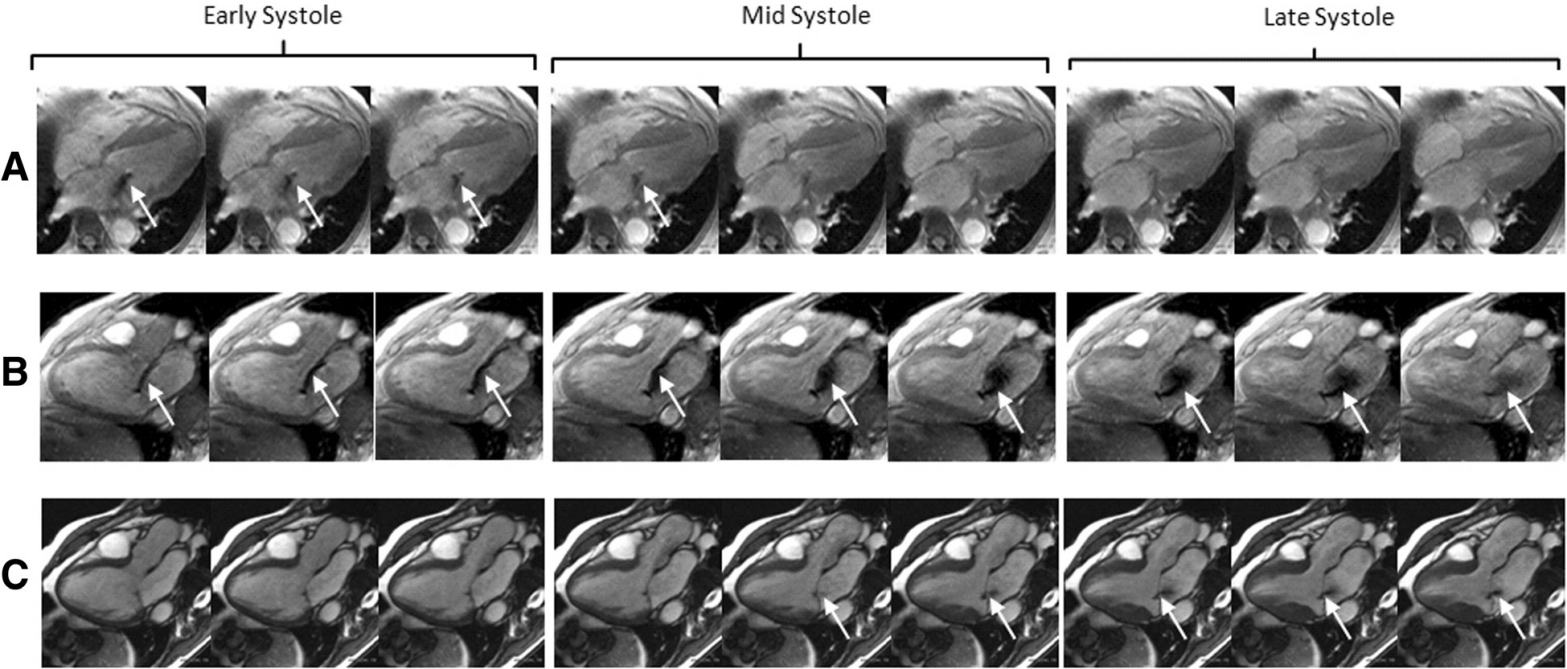
**An example of the three types of MR jet pattern. (A)** Fast cine 4 chamber view of a patient with an early MR jet pattern. The regurgitant jet is visible only during the early and mid portion of systole. **(B)** Fast cine 3 chamber view of a patient with a holosystolic MR jet pattern, The regurgitant jet is visible throughout systole. **(C)** SSFP 3 chamber view in a patient with a late MR jet pattern. The regurgitant jet is visible during the mid and late portion of systole. MR = mitral regurgitation.

To determine the temporal characteristics of the regurgitant jets the imaging plane in which the regurgitant jet is best visualized throughout systole was determined for all CMR studies. For each study the total number of systolic frames were determined and divided into 3 equal parts: early, mid, and late. A visual inspection for the presence or absence of the regurgitant jet was made for each systolic frame (Figure 1). The jet was considered present for a particular third of systole if the jet was visible for ≥ 50% of the systolic frames comprising that third of systole. Regurgitant jets were then categorized as early if the jet was present during the early and mid portion of systole only, as holosystolc if the jet was present throughout systole, and late if the jet was present during the mid and late portions of systole only.

### Determination of the instantaneous mitral regurgitant rate

The instantaneous volume of blood arriving at the proximal ascending aorta was determined using the aortic phase contrast images. The user manually defined the circumference of the aorta. An automated algorithm determined the circumference on all other phases. Manual editing of these regions of interest was performed as necessary. Using these regions of interest, the software displayed a curve of instantaneous flow vs. time. An instantaneous volume vs. time curve was created by manually integrating the flow curve. Integration was performed on a point-by-point basis using the Newton-Coates trapezoidal rule.

The instantaneous volume of blood exiting the left ventricle was determined from the short axis and a two-chamber long axis cine images. After the user defined the left ventricular apex and base, the software automatically determined the endocardial borders of the left ventricle for all cardiac phases and slices. These borders were manually edited by the operator as necessary. Based on the endocardial borders, the software displayed a curve of left ventricular volume vs. time. The instantaneous volume of blood exiting the left ventricle was displayed by the software as the slope of the left ventricular volume versus time curve.

**Figure 2:**
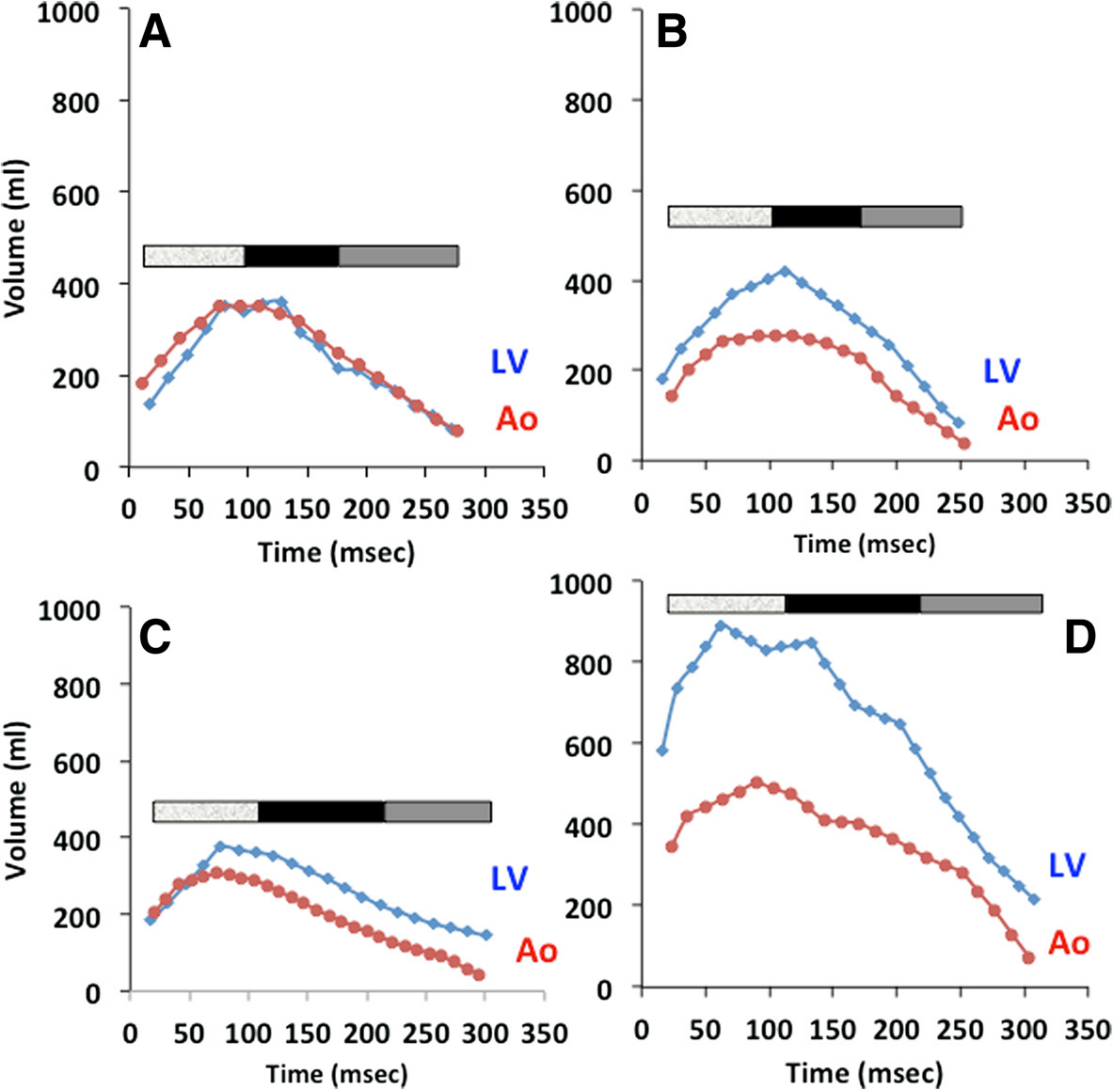
**Left ventricular stroke volume and aortic flow against time.** Systole is divided into 3 equal parts demarcated by the color bar on each graph. The mitral regurgitant jet patterns represented here are **(A)** control, **(B)** holosystolic, **(C)** late, and **(D)** early.

In patients without mitral regurgitation, the rate at which blood arrives in the proximal ascending aorta is the same as the rate at which it exits the left ventricle, due to conservation of mass (Figure 2A). In patients with mitral regurgitation, the rate of blood exiting the left ventricle exceeds the rate of its appearance in the aorta (Figure 2B-D). The magnitude of this difference for each time point is the instantaneous regurgitant rate.

In our analysis we chose to divide systole into 3 equal parts, early, mid, and late. The peak regurgitant rate was determined for each part of systole. In addition, an average regurgitatnt rate for all of systole was determined. To characterize the degree of systolic temporal variation of mitral regurgitant rate, we calculated a peak-to-average regurgitant rate ratio.

### Statistical analysis

Continuous data are presented as mean ± SD. Categorical data are presented as absolute numbers or percentages. We performed univariate analyses of continuous variables using a two-tailed Student *t* test. One-way ANOVA with a post-hoc Bonferroni test was used to compare means of continuous variables among multiple groups. All statistical analyses were performed using SPSS for Windows version 16 (SPSS Inc, Chicago, Ill). A probability value < 0.05 was considered to be statistically significant.

## Results

Baseline patient clinical characteristics and CMR data are presented in Tables 1 and 2. On CMR there were 24 (59%) patients with mild mitral regurgitation, 10 (24%) patients with moderate mitral regurgitation, and 7 (17%) patients with severe mitral regurgitation. Of the 41 patients included in this study 17 (41%) had mitral valve prolapse and 7 (17%) had a flail leaflet. The mean mitral regurgitant rates were lowest in patients with mild mitral regurgitation on CMR (79 ± 33 ml/s) and higher in those with moderate (150 ± 57 ml/s) and severe mitral regurgitation (304 ± 137 ml/s) (P < 0.0001). Similarly, the peak regurgitant rates were lowest in patients with mild mitral regurgitation on CMR (132 ± 47 ml/s) and higher in those with moderate (227 ± 93 ml/s) and severe mitral regurgitation (386 ± 135 ml/s) (P < 0.0001). There were 7 (17%) patients with an early mitral regurgitation jet pattern, 21 (51%) patients with a holosystolic mitral regurgitation jet pattern, and 13 (32%) patients with a late mitral regurgitation jet pattern.

**Table 1:** Baseline patient clinical and imaging characteristics

	**(n = 41)**
**Age, (years ± SD)**	57 ± 15
**Male, n (%)**	23 (56)
**Hypertension, n (%)**	14 (34)
**Diabetes, n (%)**	4 (10)
**Hyperlipidemia, n (%)**	11 (27)
**Smoking history, n (%)**	8 (20)
**Degenerative mitral valve disease, n (%)**	35 (85)
**Prolapse, n (%)**	17 (41)
**Flail leaflet, n (%)**	7 (17)
**Functional mitral valve, n (%)**	6 (5)
**Eccentric regurgitant jet, n (%)**	22 (54)

LV = left ventricle; RV = right ventricle.

**Table 2:** Baseline left and right ventricular indices

	**(n = 41)**
**LV end-diastolic volume (ml)**	176 ± 68
**LV end-systolic volume (ml)**	71 ± 60
**LV end-diastolic volume index (ml/m**^**2**^**)**	96 ± 32
**LV end-systolic volume index (ml/m**^**2**^**)**	39 ± 29
**LV stroke volume (ml)**	105 ± 34
**LV ejection fraction (%)**	63 ± 15
**RV end-diastolic volume (ml)**	144 ± 48
**RV end-systolic volume (ml)**	72 ± 36
**RV end-diastolic volume index (ml/m**^**2**^**)**	77 ± 20
**RV end-systolic volume index (ml/m**^**2**^**)**	39 ± 17
**RV stroke volume (ml)**	69 ± 26
**RV ejection fraction (%)**	51 ± 11

LV = left ventricular; RV = right ventricular.

**Figure 3:**
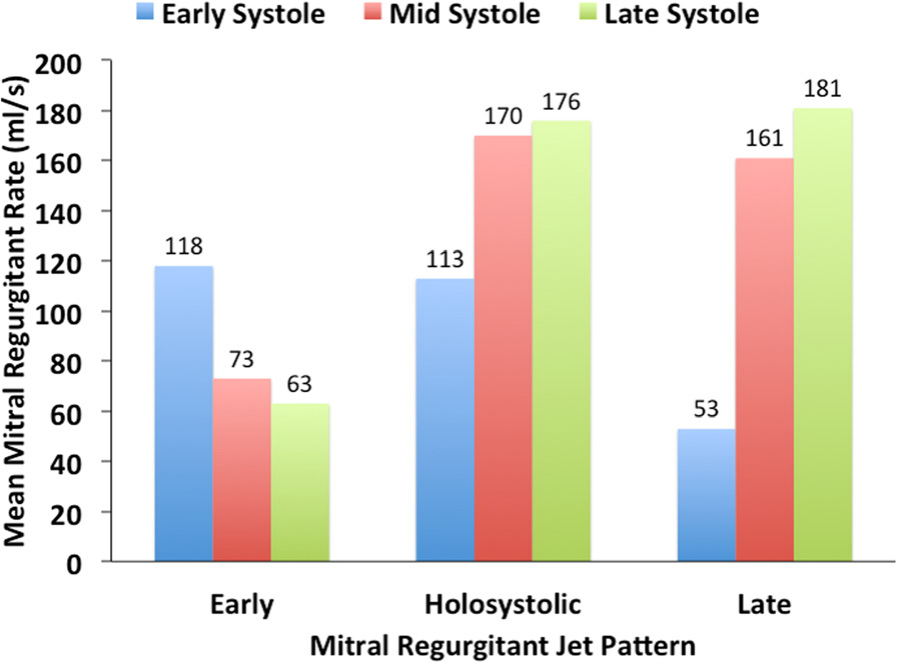
Mean mitral regurgitant rate for all three mitral regurgitant jet patterns.

**Figure 4:**
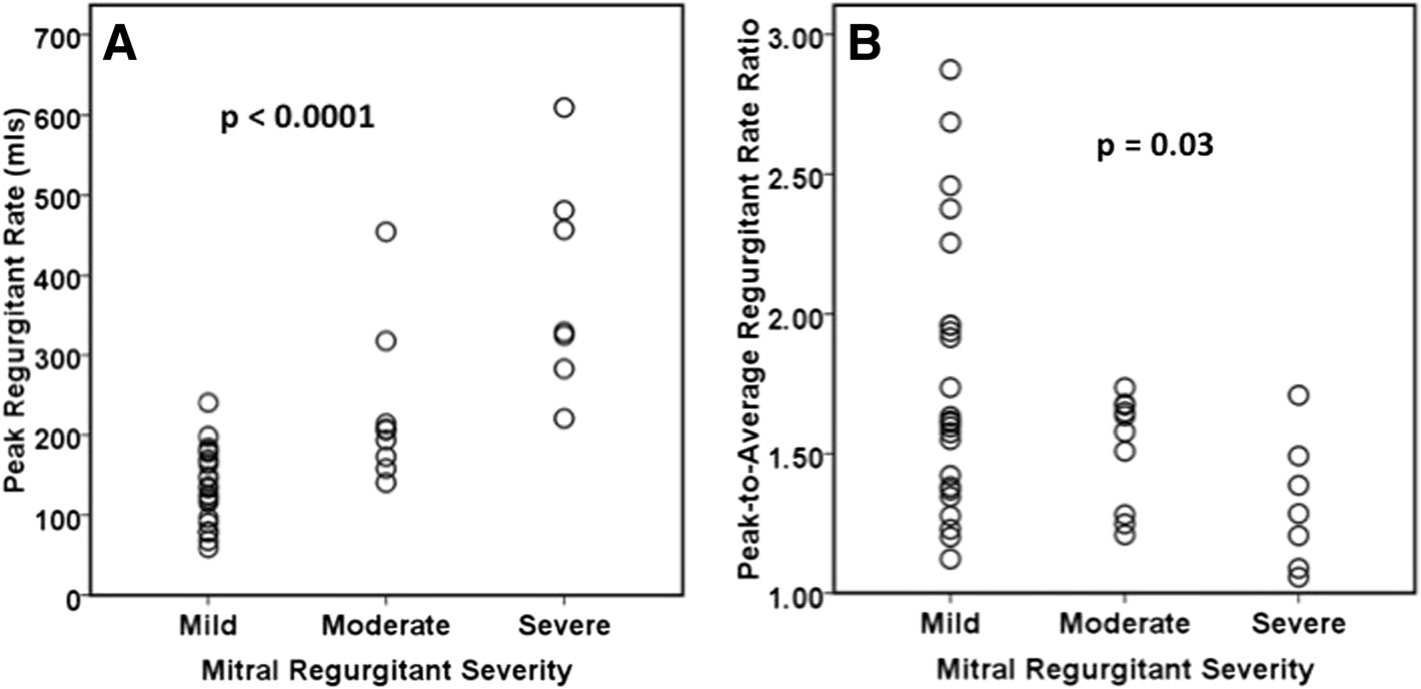
**Relationship between peak regurgitant rate and peak-to-average regurgitant rate ration and MR severity. (A)** Peak regurgitant rate for each patient according to MR severity based on CMR. **(B)** Peak-to-average regurgitant rate ratio for each patient according to MR severity based on CMR. CMR = cardiovascular magnetic resonance; MR = mitral regurgitation.

We calculated the mitral regurgitant rate for early, mid and late systole for each patient. We found that patients with early mitral regurgitation jet patterns had higher regurgitant rates in early systole, patients with holosystolic MR jet patterns had higher regurgitant rates in mid and late systole, and patients with late jet patterns had higher regurgitant rates in late systole (Figure 3). When classifying mitral regurgitation severity according to the regurgitant volume there was substantial variation of peak regurgitant rate within the mild, moderate, and severe groups (Figure 4A). This variation of peak regurgitant rate was also seen between the mitral regurgitation severity groups, even between patients with mild and severe mitral regurgitation. To assess the variation of regurgitant rate we calculated a peak-to-average regurgitant rate ratio for each patient. The peak-to-average regurgitant rate ratio was greater among patients with mild MR (1.75 ± 0.5) than those with moderate (1.52 ± 0.2) and severe (1.32 ± 0.23) MR (P = 0.03) (Figure 4B).

## Discussion

In this study we calculated the instantaneous mitral regurgitant rate using CMR. The regurgitant rate varied significantly among patients with early, holosystolic, and late jet patterns with the greatest variation among the patients with early and late patterns. Furthermore, the systolic temporal variation of the regurgitant rate was greatest among patients with mild mitral regurgitation.

Prior studies have described the systolic temporal variation of mitral regurgitant severity in a qualitative manner [4,5,7]. This temporal variation of mitral regurgitant severity is thought to be due to the interplay of the transmitral pressure and the mitral annulus size [4], both dynamic features of left ventricular function. The peak regurgitant rate is determined by several factors one of which is the transmitral pressure. One of the determinants of transmitral pressure is left ventricular systolic function. Although the peak regurgitant rate is affected by left ventricular systolic function, the temporal variation of peak regurgitant rate is not affected by left ventricular systolic function since patients would be expected to have both a low peak regurgitant rate and a low mean regurgitant rate. To illustrate this we calculated a peak-to-average rate ratio which controls for the left ventricular systolic function of each patient (Figure 4B). We found a greater variation in the peak-to-average regurgitant rate ratio in patients with mild mitral regurgitation when compared to patients with moderate or severe mitral regurgitation (Figure 4B).

To our knowledge this is the first study to quantify the systolic temporal variation of mitral regurgitation. Among all three types of jet patterns described in this study there was significant temporal variation of mitral regurgitant rate, with the greatest variation in those with early and late jet patterns. In the overall cohort, the mean and peak regurgitant rates increased with worsening mitral regurgitation severity, with the lowest regurgitant rates in patients with mild mitral regurgitation and the highest regurgitant rates in patients with severe mitral regurgitation. However, we found: 1) significant variation of theses rates within the mild, moderate, and severe groups, and 2) significant overlap of these rates between these groups. Furthermore, we found that the greatest temporal variation of regurgitant rate was among patients with mild mitral regurgitation as illustrated by the high peak-to-average regurgitant rate ratios among patients with mild mitral regurgitation.

These findings reinforce the American Society of Echocardiography recommendations to employ quantitative methods that account for the temporal variation of mitral regurgitation [2]. Parameters such as the proximal isovelocity surface area and vena contracta are most easily measured during the portion of systole when the color Doppler jet is best visualized. Our data reinforces the assertion that a measurement of mitral regurgitant severity at a point in time during systole may not be representative of the overall severity and may lead to overestimation or underestimation of mitral regurgitant volume. Hence, it is important to account for the temporal variation of mitral regurgitation as recommended by the American Society of Echocardiography [2]. The variation of regurgitant rate was greatest among patients with mild mitral regurgitation and lowest among those with severe mitral regurgitation. Thus, overestimation of mitral regurgitation based on a single measurement is most likely to occur in patients with mild or moderate mitral regurgitation. These findings highlight the advantage of using mitral regurgitant volume as a measure of mitral regurgitant severity over measures of mitral severity that are routinely made during a single point in systole, such as PISA, vena contracta, and jet width. Mitral regurgitant volume, such as measured by CMR, measures the amount or regurgitation through all of systole without reliance on a single point in systole, and thus is not susceptible to the temporal variation of mitral regurgitant severity we observed in this study.

To illustrate the possibility of overestimation due to the variation of regurgitant rate, if one is to assume a systolic period of 400 msec, a regurgitant rate of 150 ml/s throughout systole would result in severe mitral regurgitation (150 ml/s * 0.4 sec = 60 ml). In our study, a peak regurgitant rate ≥ 150 ml/s was seen in 7/17 (29%) patients with mild mitral regurgitation and 9/10 (90%) patients with moderate mitral regurgitation. Furthermore, taking a typical patient from the current study who has an early jet pattern with regurgitant rates of 178 ml/s, 39 ml/s, and 0 ml/s in early, mid, and late systole respectively; the assumption of a constant regurgitant rate throughout systole leads to a significant overestimation of mitral regurgitant volume (178 ml/s * 0.4 ms = 72 ml) compared to the actual volume of mitral regurgitation [(178 ml/s * 0.13 s) + (39 mls * 0.13 sec) + (0 ml/s * 0.13 sec) = 28 ml] for this patient.

This study has several limitations. The study population is small and the results are limited to the patient population that we studied, namely those with lone mitral regurgitation referred for evaluation of their mitral regurgitation. The calculation of regurgitant rate was partly based on velocity encoded imaging on CMR which is less accurate when velocities through the aortic valve are high.

## Conclusions

CMR can quantify the systolic temporal variation of mitral regurgitation. There is significant temporal variation of the mitral regurgitant rate even among patients with holosystolic mitral regurgitant jets, and especially among patients with mild and moderate mitral regurgitation. The findings of this study highlight the need to use quantitative measures of mitral regurgitant severity that take into consideration both the absolute regurgitant volume and the temporal variation of mitral regurgitation.

## Competing interests

The authors have no competing interests that relate to this study. Dr. Steven Wolff is the owner of NeoSoft, LLC and NeoCoil, LLC.

## Authors’ contributions

SU, SDW, LG, and FC designed the study, performed data analysis, and prepared the manuscript. AS performed statistical analysis. SG, SKB, HP, EB, RC, and NT performed patient recruitment, data collection, and analysis. All authors read and approved the final manuscript.
